# Exploring oncogenic driver molecular alterations in Hispanic/Latin American cancer patients: A call for enhanced molecular understanding

**DOI:** 10.18632/oncoscience.597

**Published:** 2024-04-22

**Authors:** Rafael Parra-Medina

**Keywords:** Latin America, Hispanic, cancer, molecular, lung

Latin America’s (LA) population is a heterogeneous mix of Amerindian, African, and Caucasian ancestries with different proportions in different regions. Countries such as Argentina, Brazil, Colombia, Costa Rica, Uruguay, and Venezuela have a higher proportion of Caucasian while regions in Mexico, Perú, and Bolivia have a higher proportion of Amerindian ancestries [1]. Although the overall incidence of cancer in Latin American countries is generally lower compared to high-income nations, whereas the mortality rate is notably higher [2]. This disparity can be attributed to several factors, including smoking habits, diet quality, levels of physical activity, access to healthcare services, and availability of cancer screening programs [3].

Recently, advancements in the understanding of oncogenic driver molecular alterations across various tumor types, including lung, breast, melanoma, colorectal, urothelial, cervical, thyroid, and brain cancers, have paved the way for targeted therapies. These therapies have notably enhanced patient outcomes. The molecular alterations included mutations, rearrangement, copy number variations, and protein expression. Diagnostic techniques like Immunohistochemistry (IHC), Fluorescence *In Situ* Hybridization (FISH), real time PCR, digital PCR, Sanger sequencing and Next-Generation Sequencing (NGS), play a crucial role in identifying these alterations, enabling more personalized and effective treatment approaches [4].

Lung cancer is the tumor with major oncogenic driver molecular alterations with treatment options. Targeted molecular alterations include *ALK* (anaplastic lymphoma receptor tyrosine kinase), *BRAF* (V-raf murine sarcoma oncogene homolog B1), *EGFR* (epidermal growth factor receptor), *HER-2* (human epidermal growth factor receptor), *KRAS* (Kirsten rat sarcoma virus), *MET* (MET proto-oncogene receptor tyrosine kinase), *NTRK* (neurotrophic tropomyosin receptor kinase), *RET* (rearranged during transfection), *ROS1* (c-ros oncogene 1), and *NRG1* (neuregulin-1) [5].

The prevalence of driver mutations in lung cancer patients exhibits variation influenced by factors such as ethnicity, smoking status, and gender [6]. A recent systematic review and metanalysis [7] aimed to ascertain the prevalence of actionable mutations specifically in the Hispanic/Latino population diagnosed with non-small cell lung cancer (NSCLC). This comprehensive analysis involved the extraction and examination of clinical data from 55 articles. The more tested genes were *EGFR* (41 articles), *ALK* (29 articles), *KRAS* (26 articles), and *ROS1* (13 articles). The tests of other genes were found in less than 10 articles. This is related to the approved targeted therapy, laboratory capability, and prevalence of the molecular alteration. Among the most important results that were observed, *EGFR* and *KRAS* were the most prevalent genes with high heterogeneity across the countries. The overall mutation frequency for *EGFR* was 22%. The most frequent mutations in the *EGFR* gene were del19 (10%) and L858R (7%). However, countries with a high proportion of Amerindian ancestries show a higher prevalence of *EGFR* in contrast to countries with a high proportion of Caucasians. The prevalence of *EGFR* varies significantly across different populations: it is estimated at 38.8% in Asian populations, 17.4% in White populations, and 17.2% in Black populations [8]. The systematic review and metanalysis [7] also found that the mean of *KRAS* mutation was a 14% prevalence. *KRASG12C* was the most frequent mutation with a 7% prevalence in an entire population. The overall frequency of *ALK* rearrangement was 5%. The mean frequency of *ROS-1* rearrangement was 2%, and the frequencies of *HER-2, MET, BRAF, RET, NTRK* molecular alterations were 4%, 3%, 2%, 2%, and 1% respectively. A major limitation of the study was the lack of information on some countries or studies with a small sample size affecting the real prevalence data. Additionally, the limited availability of NGS data in the included articles represents a significant gap compared with studies in other population.

The prevalence of molecular alterations in tumors such as breast and colorectal cancer also exhibit distinct patterns in the LA population compared to other populations [9]. Unfortunately, LA faces multiple challenges in implementing diagnostic and targeted therapies, stemming from policy constraints, infrastructural limitations, and a shortfall in human expertise [4]. In addition, the representation of LA patients in large cohort studies remains below 2%, limiting the exploration of molecular epidemiology [9].

In conclusion, this editorial underscores the complex molecular diagnosis landscape of cancer in the LA population. While advances in the understanding of oncogenic molecular alterations have led to targeted therapies improving outcomes, the diversity in this population poses unique challenges. The prevalence of mutations as lung cancer patients, for instance, varies significantly across different ethnic groups, indicating the need for tailored approaches in diagnosis and treatment. Therefore, we need to enhance molecular diagnostic, molecular research, and healthcare cancer patients access in LA is crucial for the effective management, reflecting the need for more personalized and region-specific medical interventions.
